# Predicting habitat suitability of Korean *Lindera* as Tertiary relict plants under climate change scenarios

**DOI:** 10.1371/journal.pone.0350199

**Published:** 2026-06-03

**Authors:** Jaewon Seol, Hye-jin Kwon, Songhie Jung, Yong-Chan Cho

**Affiliations:** 1 Forest Biodiversity Conservation Research Division, Korea National Arboretum, Pocheon, Republic of Korea; 2 DMZ Forest Biological Resources Research Division, Korea National Arboretum, Yanggu, Republic of Korea; MARE – Marine and Environmental Sciences Centre, PORTUGAL

## Abstract

Climate change profoundly affects plant habitats and ecological niches, particularly among Tertiary relict flora—remnants of warm and humid climatic conditions that prevailed during the Tertiary period—which are recognized as highly climate-sensitive lineages. The genus *Lindera* (Lauraceae), a representative group of deciduous broad-leaved trees in East Asian temperate forests, provides an ideal model for examining shifts in habitat suitability and changes in predicted suitable environments under future climate change scenarios. In this study, we developed ensemble species distribution models (SDMs) using six algorithms to predict the distributions of four *Lindera* species—*L. obtusiloba, L. glauca, L. erythrocarpa*, and *L. sericea*—under three Shared Socioeconomic Pathway (SSP) scenarios (SSP1–2.6, SSP3–7.0, SSP5–8.5). Among the three categories of environmental variables, climatic factors exerted the greatest influence on habitat suitability, with temperature seasonality (bio4) and growing-season precipitation (gsp) identified as the primary determinants. With intensifying climate change, suitable habitats shifted northward and upward, accompanied by pronounced habitat losses across southern and central Korea. Despite its broad geographic range, *L. obtusiloba* exhibited an 81% reduction in suitable habitat, whereas *L. sericea*, due to its localized distribution, showed a 91% decrease and was identified as the most climate-vulnerable species. Ecological niche overlap (Schoener’s *D*) declined across all scenarios, indicating increasing ecological differentiation among species. Although the four *Lindera* species exhibited distinct spatial responses, all consistently experienced range contractions and reduced overlap in predicted suitable environments, indicating high vulnerability to climate change. These results suggest that intrinsic ecological traits, climatic sensitivity, and niche stability—rather than current geographic range extent—are key determinants of species persistence. Accordingly, *Lindera* species in southern Korea should be considered climate-vulnerable taxa, and conservation strategies should integrate the protection of climatically stable refugia with complementary conservation measures beyond natural habitats to ensure long-term persistence under future climate change.

## 1. Introduction

Climate change profoundly influences the ecological niches of plant species, driving shifts in species distributions along elevational and latitudinal gradients and ultimately reshaping regional biodiversity patterns [1–4]. Species distribution models (SDMs) has been increasingly employed to project future species distributions under various climate scenarios [5–9] and to forecast large-scale biodiversity patterns [10]. Although ecological models have inherent limitations in fully representing climatic sensitivity [11], SDMs remains one of the most powerful and widely used analytical tools for evaluating habitat suitability under present and future climate conditions [12].

On the Korean Peninsula, southern temperate plants may benefit from ongoing warming, as their suitable habitats are projected to expand northward under climate change. For instance, several members of the Lauraceae—such as Machilus, Cinnamomum, and Neolitsea, which are characteristic species of evergreen broad-leaved forests—are predicted to expand throughout southern Korea [13].

In contrast, southern endemic species such as *Coreanomecon hylomeconoides* and *Stewartia koreana* are projected to face extinction across all scenarios [14,15]. These contrasting projections highlight that climatic responses can differ substantially among taxa, emphasizing the importance of species- or population-level modeling to detect shifts in biodiversity patterns and to provide spatially explicit information for prioritizing conservation targets [10].

In East Asia, members of the Lauraceae, including *Lindera*, are regarded as relict taxa that retreated to lower latitudes following the warm and humid climatic conditions of the Tertiary period and are thus considered highly sensitive to contemporary climatic change. The remaining Tertiary relict flora in temperate East Asia are characterized by disjunct distributions, climatic vulnerability, and locally high genetic diversity shaped by complex mountain topography and climatic heterogeneity [16,17]. Assessing the responses of such southern temperate plants to climate change is therefore crucial for developing conservation strategies that incorporate both ecological and genetic dimensions of relict taxa.

In this study, we focused on the genus *Lindera* (Lauraceae) which comprises four deciduous broad-leaved species in Korea: *L*. *obtusiloba*, *L. glauca*, *L*. *erythrocarpa*, and *L. sericea* [18]. Unlike evergreen Lauraceae species adapted to warmer climatic conditions, *Lindera* species are deciduous taxa that may exhibit different climatic sensitivities and responses to warming. Except for *L*. *obtusiloba*, which occurs widely across the peninsula, the remaining three species are confined mainly to the mountainous regions of southern Korea, each exhibiting distinct ecological and geographical patterns. Although *L*. *obtusiloba* has a broad distribution extending from southern China to the Russian Far East, previous studies suggest that even widely distributed temperate species may experience habitat loss under future climate scenarios [19]. These patterns indicate that southern temperate taxa such as *Lindera* may be particularly vulnerable to rapid climatic shifts.

Accordingly, understanding the spatial dynamics and diversity changes of deciduous *Lindera* species in southern Korea is essential for developing adaptive conservation strategies. Specifically, this study aimed to: (1) quantify changes in the distribution ranges and climatic sensitivity of each species; (2) identify ecological differentiation and interspecific relationships within the genus through analyses of ecological niche overlap; and (3) delineate climate-vulnerable areas and diversity distribution patterns to inform conservation planning for *Lindera* species under future climate conditions.

## 2. Methods

### 2.1. Study area

This study was conducted in southern Korea (33°–38° N, 125°–131° E), a region characterized by complex mountainous terrain and numerous islands. Such geomorphological diversity creates a wide range of environmental conditions that support diverse plant assemblages [20,21].

The area experiences a mixed continental and oceanic climate, with cold-temperate conditions in the north and warm-temperate conditions in the south, accompanied by pronounced seasonal temperature variation [22]. The Baekdudaegan mountain range serves as a central climatic and floristic divide across the peninsula, while its moderate slopes and heterogeneous topography facilitate the dispersal and migration of many plant species [23].

### 2.2. Species and occurrence data

**Fig 1:**
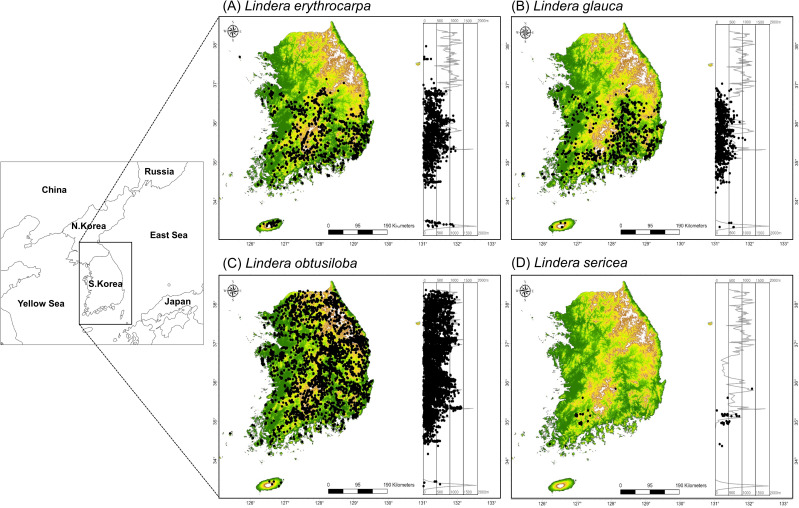
Geographic distribution and elevational range of the *Lindera* genus in South Korea. The map shows the recorded occurrences of *Lindera* species (black dots) across South Korea. Background colors represent elevation gradients (green for lowlands, yellow–brown for mid-elevations, and white for high mountains). The accompanying graph illustrates the relationship between elevation (x-axis, m) and latitude (y-axis) of *Lindera* occurrences. Geographic layers (e.g., country boundaries and coastlines) were obtained from Natural Earth (https://www.naturalearthdata.com/), which is in the public domain. The maps were generated using ArcGIS Pro version 3.2 (Esri Inc.).

The genus *Lindera* (Lauraceae) is distributed mainly across temperate and subtropical regions of East Asia. Although *Lindera* species can tolerate a broad range of environmental conditions, their habitat ranges and climatic sensitivities differ markedly among species. In particular, *L. sericea* exhibits an extremely localized distribution and is likely the most climate-sensitive species within the genus. Species occurrence data were compiled from nationwide vegetation surveys conducted between 2002 and 2023. A total of 7,011 occurrence records for *L. obtusiloba*, 1,029 for *L. glauca*, 2,088 for *L. erythrocarpa*, and 37 for *L. sericea* were collected and used for modeling analyses (Fig 1).

*Lindera obtusiloba* is a large deciduous shrub widely distributed across Korea, Japan, China, and the Russian Far East, typically inhabiting adequately moist foothill and valley forests. *L*. *glauca*, found in China, Japan, and Korea [18], generally grows in lowland and valley forests as a small deciduous tree [24,25]. *L*. *erythrocarpa* is a large deciduous tree occurring in temperate forests of Korea, Japan, and China [26]. *L*. *sericea*, native to Korea and Japan, is a large deciduous shrub that inhabits rocky slopes and humid foothills [27,28].

In Korea, *L*. *obtusiloba* has the broadest distribution, extending from northern to southern regions of the peninsula, whereas *L*. *erythrocarpa*, *L*. *glauca*, and *L*. *sericea* are largely confined to southern provinces such as Chungcheong, Jeolla, and Gyeongsang. Among these, *L*. *sericea* is a rare and geographically restricted species with an extremely narrow distribution range.

### 2.3. Selection and processing of environmental variables

Environmental variables were prepared for four time periods: the baseline period (1981–2010), near future (2011–2040), mid-future (2041–2070), and far future (2071–2100), and for three SSP scenarios (SSP1–2.6, SSP3–7.0, SSP5–8.5). An initial set of 38 variables (27 bioclimatic, 7 soil-related, and 4 topographic variables) was considered. To minimize multicollinearity among predictors, variables with pairwise Pearson correlation coefficients |*r*| ≥ 0.7 were excluded, resulting in a final set of 25 variables for modelling. All variables were standardized to a spatial resolution of 250 m. The final dataset included 14 bioclimatic variables obtained from the CHELSA dataset (v2.1; [29]; www.chelsa-climate.org), which provides baseline (1981–2010) and CMIP6-based future climate projections derived from the GFDL-ESM4 general circulation model, downscaled following the ISIMIP3b bias correction framework. In addition, eight soil-related variables were obtained from SoilGrids (www.soilgrids.org), and three topographic variables were derived from NASA SRTM 90 data (www.cmr.earthdata.nasa.gov) (S1 Table in S1 File).

### 2.4. Construction of species distribution models (SDMs)

Species distribution modeling was performed using presence–background data. For each species, 1,000 pseudo-absence points were randomly generated using the biomod2 framework. To account for large differences in occurrence sample sizes among species, records for *L. obtusiloba* were randomly subsampled to 500 prior to modelling. The number of pseudo-absences was fixed at 1,000 for all species, resulting in varying effective prevalence across species. To reduce potential spatial sampling bias, duplicate occurrence records within the same raster grid cell were removed during data formatting.

An ensemble modeling framework was implemented by combining six algorithms, which were grouped into three methodological categories: (1) Machine-learning methods: Gradient Boosting Machine (GBM; [30]), Random Forest (RF; [31]), and Artificial Neural Network (ANN; [32]); (2) Regression-based methods: Multivariate Adaptive Regression Splines (MARS; [33]) and Generalized Additive Model (GAM; [34]); and (3) Classification-based method: Classification Tree Analysis (CTA; [35]). This combination was selected to represent diverse methodological approaches, as ensemble modelling across multiple algorithms has been shown to improve predictive performance [36,37].

Model performance was evaluated using three accuracy metrics: Cohen’s Kappa, True Skill Statistic (TSS), and the area under the receiver operating characteristic curve (AUC) (S2 Table in S1 File). Model calibration and evaluation were performed using repeated random cross-validation with a 70/30 split between calibration and evaluation datasets across three replicates. Ensemble models were constructed using three ensemble approaches implemented in biomod2: coefficient of variation (EMcv), confidence interval (EMci), and weighted mean (EMwmean). Only models with AUC values ≥ 0.7 were retained for ensemble modeling [38] (S3 Table in S1 File). Variable importance was assessed using a permutation-based approach implemented in the biomod2 ensemble framework, which evaluates the decrease in model performance when the values of each predictor are randomly permuted. Importance values were derived from the weighted mean ensemble model (EMwmean, range 0–1) and interpreted descriptively as relative contributions of each predictor.

### 2.5. Evaluation of geographic shifts and climatic sensitivity

The biogeographical characteristics of the four *Lindera* species were analyzed based on their occurrence frequencies across vegetation climate zones and floristic regions in Korea. The Korean Peninsula was divided into three vegetation climate zones—northern temperate, central temperate, and southern temperate—and four floristic regions: I (cold), II (cool), III (warm), and IV (maritime) [39].

Geographic shifts in latitude, longitude, and elevation were quantified using the predicted maps generated from ensemble SDMs under each climate change scenario. Suitable habitats were defined using the threshold that maximizes the True Skill Statistic (TSS), as implemented in the biomod2 framework. To minimize the influence of outliers, the median rather than the mean was used when calculating changes in latitude, longitude, and elevation [40]. These analyses were conducted consistently across all three SSP scenarios (SSP1–2.6, SSP3–7.0, and SSP5–8.5).

Climatic sensitivity to climate change was assessed by calculating the proportional change in suitable habitat area (increase or decrease). For this purpose, the Range Change Index (RCI) was computed, representing the relative net change in suitable area compared with the baseline distribution [41]. A positive RCI indicates an expansion of suitable habitat, whereas a negative RCI indicates contraction. For example, RCI = –100 denotes complete habitat loss within the study area. The RCI was calculated as:


$$\begin{array}{c} \begin{array}{c} Range\;change\;index=\frac{S_{colonization}-\;S_{extinction}}{S_{present\;range\;area}}\times \text{1}\text{0}\text{0}\; \end{array} \end{array}$$ (1)

where, $S_{colonization}$ is the area of newly suitable habitat, $S_{extinction}$ is the area of lost habitat, and $S_{present\;range\;area}$ is the baseline suitable habitat area.

### 2.6. Ecological niche overlap analysis

To quantify overlap in predicted suitable habitat among the four *Lindera* species, Schoener’s *D* index was applied [42]. Overlap between each pair of species was calculated by comparing binary habitat suitability rasters derived from ensemble model outputs. Schoener’s *D* measures the degree of overlap in predicted suitable habitat between two species on a scale from 0 to 1, where values near 0 indicate minimal overlap in suitable conditions and values near 1 indicate high similarity in predicted suitable habitat [41–43]. The analysis was performed for both the baseline period and the far-future period (2071–2100) under three climate change scenarios (SSP1–2.6, SSP3–7.0, and SSP5–8.5).

To characterize spatial shifts in habitat suitability, geographic centroids of predicted suitable habitat and the Range Change Index (RCI) were also calculated for each species and scenario (Fig 6). All analyses were conducted in R version 4.3.0 [44]. Species distribution modeling (SDMs) was performed using the biomod2 package [37], and overlap was quantified using Schoener’s *D* implemented in the dismo package [45].

## 3. Results

### 3.1. Biogeographic characteristics

**Fig 2:**
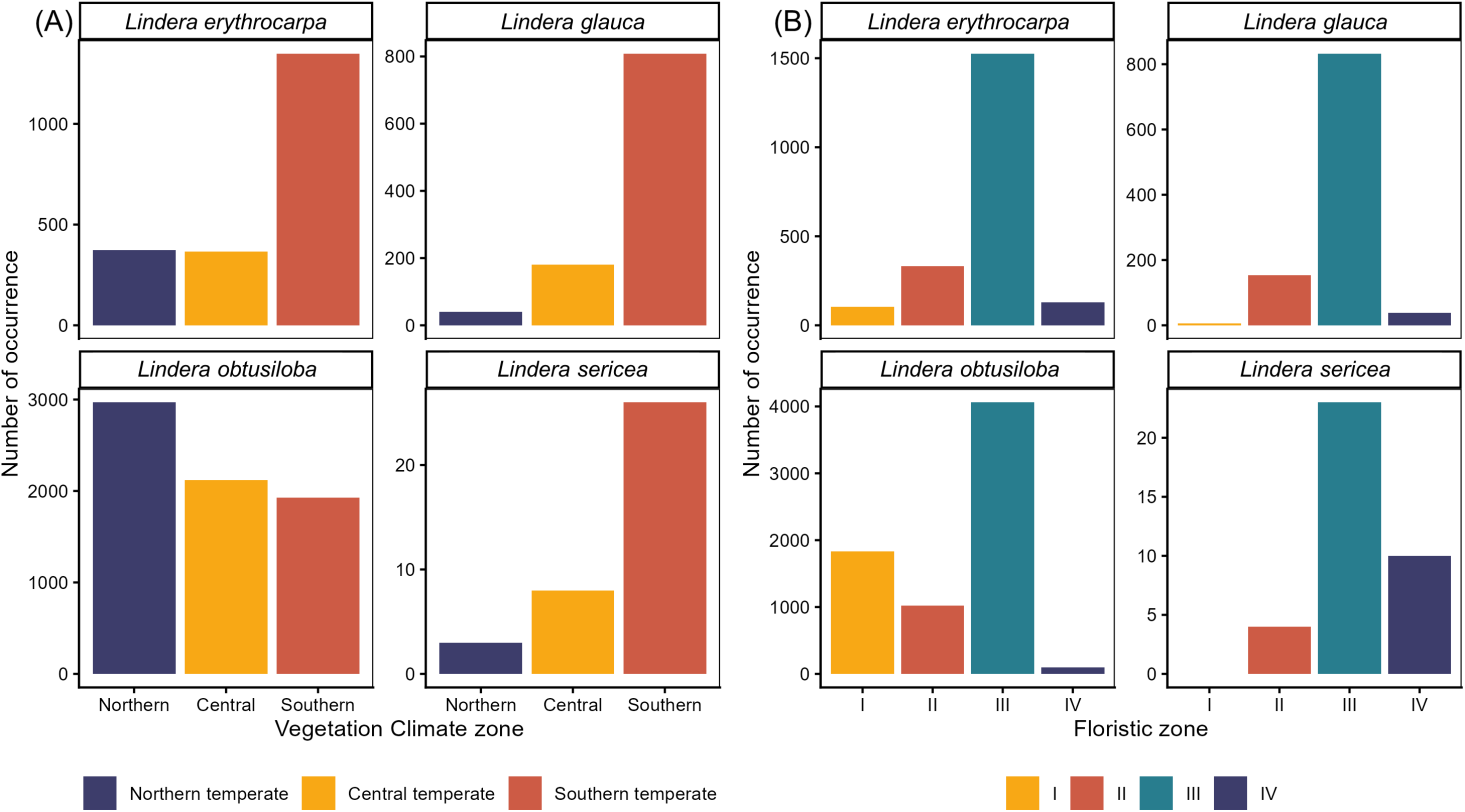
Biogeographic occurrence patterns of *Lindera* species across vegetation and floristic zones. (A) Number of occurrences in each vegetation climate zone: Northern temperate, Central temperate, and Southern temperate. (B) Number of occurrences in each floristic zone: I (cold, high-elevation midland areas), II (cool, high-elevation southern areas), III (warm, hilly middle and southern regions), and IV (maritime coastal and island zones).

*Lindera erythrocarpa* (64.6%), *L*. *glauca* (78.5%), and *L*. *sericea* (70.3%) exhibited their highest occurrence frequencies within the southern temperate vegetation climate zone, whereas *L. obtusiloba* showed the highest frequency (42.3%) in the northern temperate zone (Fig 2A). Except for *L*. *obtusiloba*, the other three species displayed an increasing trend in occurrence frequency from the northern to the southern temperate zones.

Across floristic regions, all four species recorded their highest occurrences in the warm floristic zone (Zone III): *L*. *erythrocarpa* (73.0%), *L. glauca* (80.9%), L*. obtusiloba* (57.9%), and *L*. *sericea* (62.2%) (Fig 2B). In contrast to the other species, *L*. *sericea* was absent from the maritime floristic zone (Zone IV).

### 3.2. Relative contribution of environmental variables

Model performance was evaluated using the True Skill Statistic (TSS), the Area Under the Receiver Operating Characteristic Curve (ROC), and Cohen’s Kappa. The models showed good predictive performance across algorithms, with mean TSS values ranging from 0.68 to 0.74 and ROC values exceeding 0.87 (Supplementary S2 and S3 Tables in S1 File).

The results of SDMs indicated that climatic variables showed high relative importance across *Lindera* species (Table 1). Among the four species, temperature seasonality (bio4) showed the highest relative importance for *L. erythrocarpa* (0.35) and *L. obtusiloba* (0.45), both of which are widely distributed across southern Korea. For *L. glauca*, isothermality (bio3, 0.13) had the highest relative importance. For *L. sericea*, growing-season precipitation (gsp, 0.25) had the highest relative importance, followed by temperature seasonality (bio4, 0.18) and surface solar radiation (srad, 0.10).

**Table 1:** Relative importance of environmental variables in SDMs. Values are expressed as permutation-based importance (0–1). Bold values indicate the most influential variable for each species.

Order	Abbreviation	Variable Class	Explanation	Contribution (%)			
				*L.* *erythrocarpa*	*L.* *glauca*	*L.* *obtusiloba*	*L.* *sericea*
**1**	**bio2**	Climate	Mean diurnal air temperature range	0.09	0.07	0.02	0.03
**2**	**bio3**	Climate	Isothermality (bio2/bio7)(×100)	0.09	**0.13**	0.02	0.05
**3**	**bio4**	Climate	Temperature Seasonality (standard deviation ×100)	**0.35**	0.07	**0.45**	**0.18**
**4**	**bio13**	Climate	Precipitation amount of the wettest month	0.02	0.01	0.00	0.02
**5**	**bio14**	Climate	Precipitation amount of the driest month	0.00	0.00	0.01	0.04
**6**	**bio15**	Climate	Precipitation seasonality	0.02	0.01	0.01	0.02
**7**	**gsp**	Climate	Precipitation sum accumulated on all days during the growing season based on TREELIM [46]	0.01	0.00	0.04	**0.25**
**8**	**gst**	Climate	Mean temperature of all growing season days based on TREELIM	0.01	0.00	0.00	0.01
**9**	**kg0**	Climate	Köppen-Geiger climate classification	0.00	0.03	0.00	0.00
**10**	**kg2**	Climate	Modified Köppen-Geiger climate classification 1	0.00	0.00	0.00	0.01
**11**	**kg3**	Climate	Modified Köppen-Geiger climate classification 2	0.00	0.00	0.00	0.00
**12**	**kg4**	Climate	Modified Köppen-Geiger climate classification 3	0.00	0.01	0.00	0.00
**13**	**kg5**	Climate	Modified Köppen-Geiger climate classification 4	0.00	0.00	0.01	0.01
**14**	**npp**	Climate	Net primary productivity	0.00	0.01	0.03	0.09
**15**	**bdod**	Soil	Bulk density of the fine earth fraction	0.00	0.00	0.01	0.0
**16**	**cfvo**	Soil	Volumetric fraction of coarse fragments (>2 mm)	0.01	0.01	0.01	0.01
**17**	**ocd**	Soil	Organic carbon density	0.01	0.01	0.00	0.01
**18**	**phh2o**	Soil	Soil pH	0.01	0.00	0.05	0.01
**19**	**sand**	Soil	Proportion of sand particles (> 0.05/0.063 mm) in the fine earth fraction	0.00	0.00	0.00	0.00
**20**	**silt**	Soil	Proportion of silt particles (≥0.002 mm and ≤ 0.05/0.063 mm) in the fine earth fraction	0.00	0.00	0.01	0.01
**21**	**soc**	Soil	Soil organic carbon content in the fine earth fraction	0.01	0.01	0.08	0.02
**22**	**wavg**	Topographic	Topographic wetness index (TWI)	0.00	0.00	0.00	0.01
**23**	**elev**	Topographic	Elevation	0.01	0.00	0.00	0.01
**24**	**rough**	Topographic	Surface roughness	0.00	0.01	0.01	0.01
**25**	**srad**	Topographic	Surface solar radiation	0.01	0.01	0.01	0.10

### 3.3. Predicted changes in suitable habitats and diversity distribution

**Fig 3:**
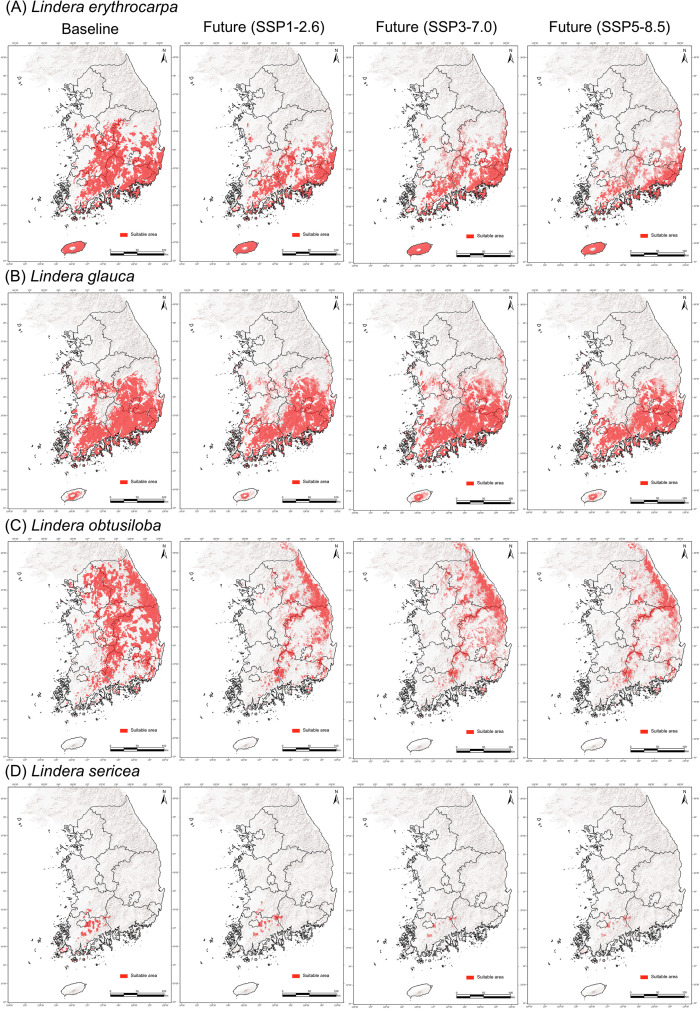
Predicted suitable habitats of four *Lindera* species under baseline and future climates. Suitable habitats were defined using the threshold that maximizes the True Skill Statistic (TSS). Binary maps were generated for each species under three SSP scenarios (SSP1–2.6, SSP3–7.0, SSP5–8.5), averaged across near-future (2011–2040), mid-future (2041–2070), and far-future (2071–2100) periods. (A) *L*. *erythrocarpa*, (B) *L*. *glauca*, (C) *L*. *obtusiloba*, (D) *L*. *sericea*.

The baseline suitable habitat of *L*. *erythrocarpa* was predicted primarily in the southern provinces—Chungcheong, Jeolla, Gyeongsang, and Jeju Island. Under all future scenarios, its suitable area was projected to decrease markedly, particularly in central and southern inland regions (Fig 3A). For *L*. *glauca*, baseline suitable habitats were also concentrated in Chungcheong, Jeolla, and Gyeongsang provinces, but were predicted to shrink substantially with increasing climate-change severity across the SSP1–2.6 to SSP5–8.5 scenarios (Fig 3B).

*L*. *obtusiloba* currently occupies almost the entire Korean Peninsula; however, in all future projections, its suitable habitats are expected to contract significantly, persisting mainly along the Taebaek and Sobaek mountain ranges (Fig 3C). The baseline distribution of *L*. *sericea* is centered around Mt. Mudeung and other mountainous areas in southern Jeolla Province, but under all climate-change scenarios, its suitable habitats are projected to decline drastically (Fig 3D).

**Fig 4:**
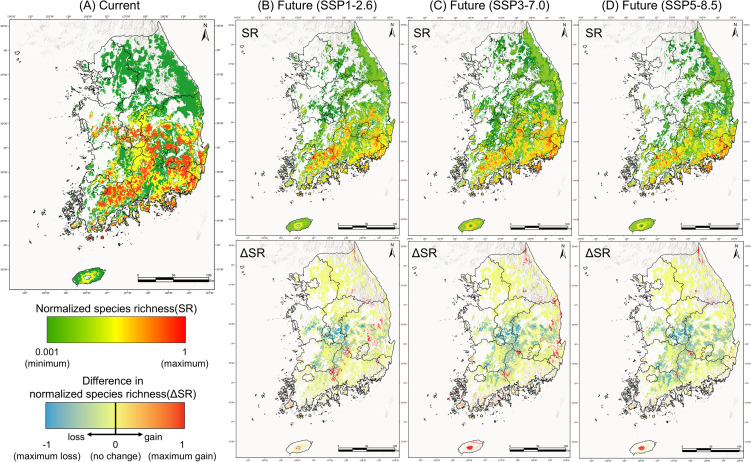
Projected spatial changes in species richness (SR) of the *Lindera* genus under SSP scenarios. Maps illustrate normalized species richness (SR; upper panels) and the difference in SR (ΔSR; lower panels) between the baseline period (1981–2010) and future climate scenarios. Background shading represents terrain variation to improve visual interpretation. (A) Baseline period, (B) SSP1–2.6, (C) SSP3–7.0, and (D) SSP5–8.5. The SR scale (green–red) represents relative species richness, while the ΔSR scale (blue–red) indicates the magnitude and direction of change. Geographic layers (e.g., country boundaries and coastlines) were obtained from Natural Earth (https://www.naturalearthdata.com/), which is in the public domain. The maps were generated using ArcGIS Pro version 3.2 (Esri Inc.).

Species richness of the genus *Lindera* is currently highest in the Chungcheong, Jeolla, and Gyeongsang regions, whereas Gyeonggi and Gangwon Provinces—dominated by *L*. *obtusiloba*—exhibit relatively low richness (Fig 4, Baseline). Under SSP1–2.6, the diversity “hotspot” contracted toward southern Jeolla and southern Gyeongsang, and under SSP5–8.5, it became even smaller, remaining only in isolated mountain refugia such as Jirisan and Mudeungsan (Fig 4, Future).

Regions of diversity decline were mainly located in northern Jeolla, northern Gyeongsang, and Chungcheong, and under SSP5–8.5, diversity loss extended across most of southern Korea (Fig 4, ΔSR, blue shades). In contrast, areas showing stable or increasing richness were primarily located in mountainous regions of southern Korea. At the species level, projected habitat reductions ranged from 13% to 91% across scenarios and time periods (Table 2 and S5 Fig in S1 File).

**Table 2:** Changes in sensitivity and suitable site area of the genus *Lindera* under different climate scenarios (SSP1–2.6, SSP3–7.0, SSP5–8.5). The table shows the projected changes in climate sensitivity and suitable habitat area (km²) for *Lindera* species across three future time periods: Near Future (2011–2040), Mid Future (2041–2070), and Far Future (2071–2100).

Species	Scenarios	Sensitivity			Surface changes(km^2^)		
		2011–2040	2041–2070	2071–2100	2011–2040	2041–2070	2071–2100
***L. erythrocarpa***	**SSP1–2.6**	–34	–32	–53	–9,660	–8,933	–14,927
	**SSP3–7.0**	–43	–15	–48	–12,277	–4,370	–13,628
	**SSP5–8.5**	–64	–26	–68	–18,109	–7,409	–19,168
***L. glauca***	**SSP1–2.6**	–13	+2	–26	–3,721	+619	–7,539
	**SSP3–7.0**	–15	**+20**	–30	–4,493	+5,896	–8,790
	**SSP5–8.5**	–29	–17	–32	–8,570	–4,981	–9,496
***L. obtusiloba***	**SSP1–2.6**	–37	–59	–68	–13,080	–20,801	–23,932
	**SSP3–7.0**	–36	–46	–76	–12,867	–16,197	–26,815
	**SSP5–8.5**	–57	–56	–81	–20,180	–19,736	–28,770
***L. sericea***	**SSP1–2.6**	–34	–35	–44	–541	–560	–689
	**SSP3–7.0**	–72	–62	**–91**	–1,132	–986	–1,445
	**SSP5–8.5**	–58	–76	–77	–915	–1,200	–1,210

### 3.4. Geographic shifts and climatic sensitivity (RCI)

**Fig 5:**
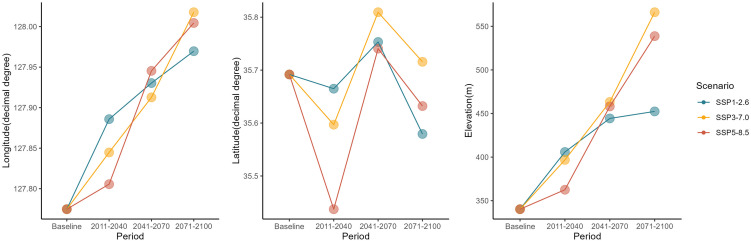
Temporal shifts in the geographic centroid of *Lindera* genus distributions under SSP scenarios. Changes in the centroid position of *Lindera* distributions are shown for longitude (A), latitude (B), and elevation (C) across four time periods (baseline, 2011–2040, 2041–2070, and 2071–2100). Lines represent different climate scenarios: SSP1–2.6 (blue), SSP3–7.0 (orange), and SSP5–8.5 (red). Each point indicates the mean centroid value for the corresponding period and scenario.

To examine geographic shifts associated with habitat contraction, the average displacement of the centroid of suitable habitats for the genus *Lindera* was calculated. Across all scenarios, the median latitude and elevation of suitable habitats increased relative to the baseline period, whereas longitude decreased under SSP3–7.0 and showed a slight increase under the other scenarios (Fig 5).

**Fig 6:**
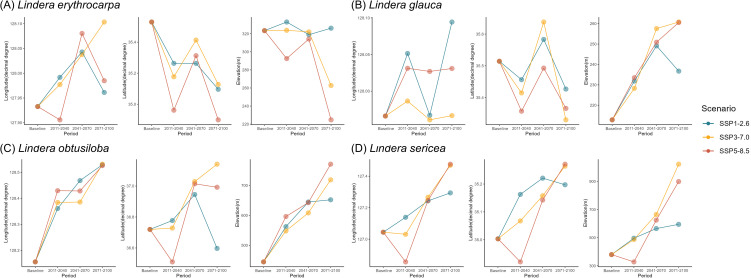
Species-specific shifts in distribution centroids of *Lindera* species under SSP scenarios. Each panel displays predicted centroid shifts (longitude, latitude, elevation) for individual species under SSP1–2.6, SSP3–7.0, and SSP5–8.5 scenarios across four time periods: baseline, 2011–2040, 2041–2070, and 2071–2100.

At the species level, all four species exhibited a northward shift in their distribution centroids by the end of the century (2071–2100) (Fig 6). Elevation increased under all scenarios except for *L. erythrocarpa*, which showed a decline under SSP3–7.0 and SSP5–8.5. Overall, all species demonstrated a consistent tendency to shift northward and toward higher elevations in response to future climate change.

The Range Change Index (RCI) analysis revealed consistent habitat loss across all four *Lindera* species, with the magnitude of loss increasing in proportion to scenario severity (Table 2). Among the species, *L*. *obtusiloba*—the most widely distributed taxon—showed the largest average reduction in suitable habitat, reaching 81% under SSP5–8.5 (2071–2100) (S5 Fig in S1 File). The greatest proportional decline was observed in *L*. *sericea*, which experienced severe habitat loss under all scenarios and time periods, with a maximum reduction of –91% under SSP5–8.5 (2071–2100). By contrast, *L*. *glauca* exhibited relatively higher resistance, showing a temporary 20% increase under SSP3–7.0 (2011–2040), followed by a 30% loss under SSP5–8.5 (2071–2100) (Table 2 and S5 Fig in S1 File).

### 3.5. Ecological niche overlap

**Fig 7:**
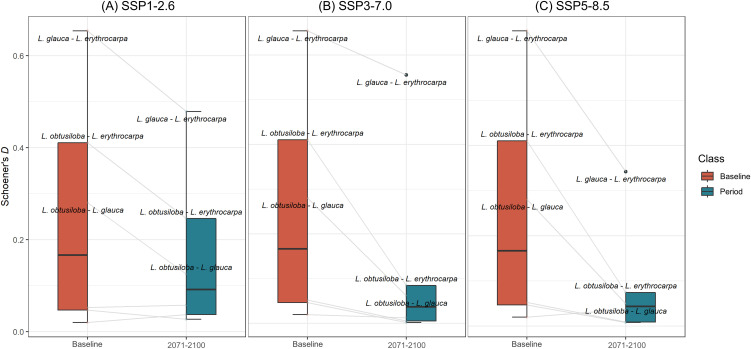
Changes in niche overlap among four *Lindera* species under SSP scenarios. Niche overlap is quantified by Schoener’s *D* (0 = no overlap; 1 = complete overlap). Panels compare baseline with the far-future period (2071–2100) for (A) SSP1–2.6, (B) SSP3–7.0, and (C) SSP5–8.5. Labels are shown only for species pairs with maximum Schoener’s *D* values exceeding 0.1.

The average ecological niche overlap (Schoener’s *D*) between the baseline and far-future periods (2071–2100) decreased across all three SSP scenarios (Fig 7). Under baseline conditions, niche overlap was highest between *L*. *glauca* and *L. erythrocarpa* (*D* = 0.654), followed by *L*. *obtusiloba*–*L*. *erythrocarpa* (*D* = 0.411) and *L*. *obtusiloba*–*L*. *glauca* (*D* = 0.281). Species pairs including *L*. *sericea* exhibited very low overlap values: *L*. *erythrocarpa*–*L*. *sericea* (*D* = 0.052), *L*. *glauca*–*L*. *sericea* (*D* = 0.047), and *L*. *obtusiloba*–*L*. *sericea* (*D* = 0.020).

Under SSP1–2.6, *L*. *glauca*–*L*. *erythrocarpa* decreased to *D* = 0.479 (–26.8%), *L*. *obtusiloba*–*L. erythrocarpa* to *D* = 0.246 (–40.1%), and *L*. *obtusiloba*–*L*. *glauca* to *D* = 0.125 (–55.5%). Under SSP3–7.0, these values were *D* = 0.556 (–15.0%), *D* = 0.085 (–79.3%), and *D* = 0.062 (–77.9%), respectively; and under SSP5–8.5, *D* = 0.342 (–47.7%), *D* = 0.074 (–81.9%), and *D* = 0.050 (–82.2%). Overall, niche overlap consistently declined with increasing climate-change intensity. An exception was observed for the *L*. *glauca*–*L*. *erythrocarpa* pair, which showed a slight increase from SSP1–2.6 to SSP3–7.0 before declining again under SSP5–8.5 (S6 Fig in S1 File).

## 4. Discussion

### 4.1. Species-specific climatic responses

All four *Lindera* species showed contractions of suitable habitats under projected climate scenarios, with differences in the magnitude and spatial pattern of their responses. Among the examined environmental variables, climatic factors exerted the strongest influence on habitat suitability, highlighting the dominant role of temperature-related variables in shaping species distributions [47].

*Lindera erythrocarpa* showed the highest relative importance for temperature seasonality (bio4) (S1 Fig in S1 File), suggesting that its distribution may be influenced by multiple climatic gradients rather than a single dominant factor [47,48]. The species’ association with mid-elevation slopes and moderately warm–humid conditions may indicate a degree of ecological flexibility within southern temperate forests.

*L. glauca* showed the highest relative importance for isothermality (bio3) (S2 Fig in S1 File), which may reflect an association with relatively stable thermal conditions. Although it may tolerate a range of climatic conditions, projected warming and increasing temperature variability may affect its future distribution, particularly in low-elevation areas.

*L. obtusiloba* showed the highest relative importance for temperature seasonality (bio4) (S3 Fig in S1 File), consistent with its broad distribution across the Korean Peninsula. However, a wide distribution does not necessarily imply climatic resilience, as species occupying diverse environments may also experience increased exposure to climatic variability. The projected reduction in suitable habitats is consistent with previous studies that have identified *L. obtusiloba* as sensitive to climate change in East Asia [19].

In contrast, *L. sericea* showed the highest relative importance for growing-season precipitation (gsp), followed by temperature seasonality (bio4) (S4 Fig in S1 File), suggesting an association with humid climatic conditions and relatively stable temperature regimes. However, these results should be interpreted with caution, as the limited occurrence data reflect the naturally restricted distribution of this species. These patterns should be interpreted within the comparative framework applied in this study.

Overall, the predicted climatic responses of *Lindera* species suggest an association with warm–humid environmental conditions. This pattern supports previous studies emphasizing that Tertiary relict flora in East Asia are highly sensitive to thermal and hydrological fluctuations [16,49]. These results further suggest that *L. sericea* may be more vulnerable to climatic instability, as its distribution appears to be associated with relatively humid and thermally stable environments.

### 4.2. Range shifts and climate-change sensitivity

Although the *Lindera* species occupy distinct ecological niches, all are projected to undergo range contractions accompanied by shifts in suitable habitats toward higher elevations and variable latitudinal changes depending on scenario and time period, likely reflecting species-specific responses to interacting temperature and precipitation gradients under different climate scenarios. As climate warming intensifies, southern and low-elevation regions are expected to become increasingly unsuitable, whereas potential refugia may persist in mountainous areas of central and northern Korea. Longitudinal shifts likely reflect the spatial distribution of major mountain ranges in South Korea, where higher elevations occur predominantly in the eastern part of the peninsula, indicating that these longitudinal shifts are closely linked to altitudinal gradients, as species track cooler conditions available in higher-elevation mountainous regions predominantly located in the eastern part of the peninsula. These upslope shifts, combined with projected reductions in suitable habitat area, are consistent with the mountain-top trap dynamic, whereby species tracking cooler climatic conditions toward higher elevations face progressively reduced habitat availability near upper elevational limits, potentially increasing extinction risk [50,51].

These results demonstrate that a broad distribution does not necessarily confer climatic resilience. For instance, *L*. *obtusiloba*—despite its wide geographic range—exhibited the largest mean decrease (–81%) in suitable habitat, indicating that even widespread taxa can remain highly vulnerable to climate change [41]. Conversely, narrowly distributed species such as *L*. *sericea* experienced severe yet spatially concentrated habitat losses, reflecting limited adaptive capacity to shifts in temperature and moisture regimes. Interestingly, *L. erythrocarpa* did not exhibit a consistent upslope shift over time, unlike the other *Lindera* species (Fig 6A). Instead, centroid elevation showed contrasting trajectories among SSP scenarios, suggesting that this species may respond to climate change primarily through spatial redistribution of suitable habitats rather than uniform upward shifts (Fig 3A).

The projected habitat suitability maps indicate relatively high stability for *L. erythrocarpa* and *L. glauca* on Jeju Island across all climate scenarios. This pattern contrasts with the substantial habitat losses projected across mainland Korea. Jeju Island has been suggested as a potential glacial refugium for warm-temperate plant species in East Asia due to its oceanic climate and complex topography [52]. These environmental characteristics may buffer climatic extremes and allow suitable habitats to persist even under future warming scenarios. Consequently, Jeju Island may function as an important climatic refugium for *Lindera* species under climate change, highlighting its potential significance for long-term conservation planning. Although such patterns may partly reflect model-related uncertainties, the consistent stability observed across scenarios suggests that this pattern is likely ecologically meaningful.

High-elevation regions, particularly the Taebaek and Sobaek mountain ranges, are likely to serve as long-term refugia that provide stable habitats for conserving genetic diversity and facilitating altitudinal migration [19]. The persistence of *Lindera* populations in these refugia will depend not only on local microclimatic stability but also on landscape connectivity among potential migration corridors [53]. Because *Lindera* species produce fleshy drupes that are dispersed by frugivorous birds [54], habitat fragmentation may limit their ability to track shifting climatic conditions through dispersal. Although genus-level assessments of climate-change impacts on *Lindera* remain limited, previous studies indicate that species within this genus are sensitive to climatic and environmental gradients. Strong genetic discontinuities have been reported in *L. obtusiloba* across East Asia, suggesting that historical climatic fluctuations have played an important role in shaping its distribution and population structure [19]. In addition, studies on other *Lindera* species have shown that local environmental conditions can strongly influence their persistence; for instance, biomass accumulation in the endangered shrub *L. melissifolia* is affected by light availability and soil flooding regimes [55]. These findings support the projected vulnerability of *Lindera* species to ongoing climatic change. The projected range contractions highlight the importance of identifying and preserving climate refugia within increasingly unsuitable landscapes.

Overall, although the trajectories of range shifts vary among species, all four *Lindera* taxa can be regarded as climate-vulnerable groups. Conservation planning should prioritize climatically stable and topographically heterogeneous areas that can function as refugia under future climate scenarios. Such mountainous regions may buffer climatic stress by maintaining locally suitable conditions and facilitating altitudinal migration. In addition, the current climatic zone associations of *Lindera* species (Fig 2) suggest that southern temperate taxa may be particularly vulnerable to warming, highlighting the importance of monitoring potential shifts in vegetation climate zones when identifying future conservation priorities.

### 4.3. Ecological differentiation under climate change

The analysis of ecological niche overlap (Schoener’s *D*) revealed a consistent decline across all future scenarios, indicating increasing ecological differentiation within the genus *Lindera*. The relatively high baseline niche overlap between *L*. *obtusiloba* and *L*. *erythrocarpa* (*D* = 0.41) reflects their shared environmental tolerances, whereas the extremely low overlap involving *L*. *sericea* (*D* < 0.05) suggesting relatively distinct environmental characteristics and a narrower ecological distribution pattern.

The progressive decrease in niche overlap under intensified climate change suggests increasing environmental segregation among species. However, because niche overlap metrics do not directly reflect interspecific interactions, further studies examining competitive dynamics and species interactions would be necessary to determine whether reduced niche overlap translates into weakened competition. This pattern is broadly consistent with theoretical predictions that climate-induced environmental filtering can promote niche divergence among closely related taxa [41,56]. The growing segregation of ecological niches among *Lindera* species underscores the need to incorporate multi-species perspectives into conservation planning. Management strategies should focus on securing areas that still support co-occurrence where niche overlap remains possible, while ensuring the long-term survival of species with distinct climatic requirements. By integrating niche-based modeling with landscape-scale connectivity analyses, future research can more effectively identify priority areas for maintaining *Lindera* diversity and enhancing the resilience of temperate forest ecosystems under changing climates.

## 5. Conclusion

This study comprehensively evaluated the effects of climate change on the habitat suitability and ecological niches of four *Lindera* species in Korea. Ensemble species distribution models (SDMs) projected northward and upward shifts in suitable habitats, accompanied by substantial habitat losses in southern and central regions.

Across all scenarios, the consistent contraction of suitable habitats and the decline in ecological niche overlap suggest that *Lindera* species will experience increasing ecological segregation and vulnerability under future climate conditions. The genus as a whole displayed a pronounced trend toward range contraction and reduced overlap in predicted suitable environments, reflecting the high climatic sensitivity characteristic of East Asian Tertiary relict flora.

These findings highlight that intrinsic ecological traits, climatic sensitivity, and niche stability—rather than the extent of current distribution—are the key determinants of long-term persistence in *Lindera* species. Accordingly, conservation planning should prioritize the identification of climatically stable refugia and the maintenance of ecological connectivity along potential migration corridors. Integrating spatial modeling with genetic diversity assessments and long-term ecological monitoring will be essential to safeguard *Lindera* diversity and the resilience of temperate forest ecosystems under accelerating climate change.

## Supporting information

S1 File Supporting figures and tables including response curves, projected suitable habitat changes, niche overlap heatmaps, environmental variables, ensemble threshold statistics, and predictive performance metrics for the four *Lindera* species. (DOCX)
