# Supplementary material for: Predicting habitat suitability of Korean Lindera as Tertiary relict plants under climate change scenarios
Source: PLoS One. 2026 Jun 3;21(6):e0350199. doi: 10.1371/journal.pone.0350199 (PMC13232808; doi:10.1371/journal.pone.0350199)
Supplement: S1 File — (DOCX) [file pone.0350199.s001.docx]

# Supporting information

**
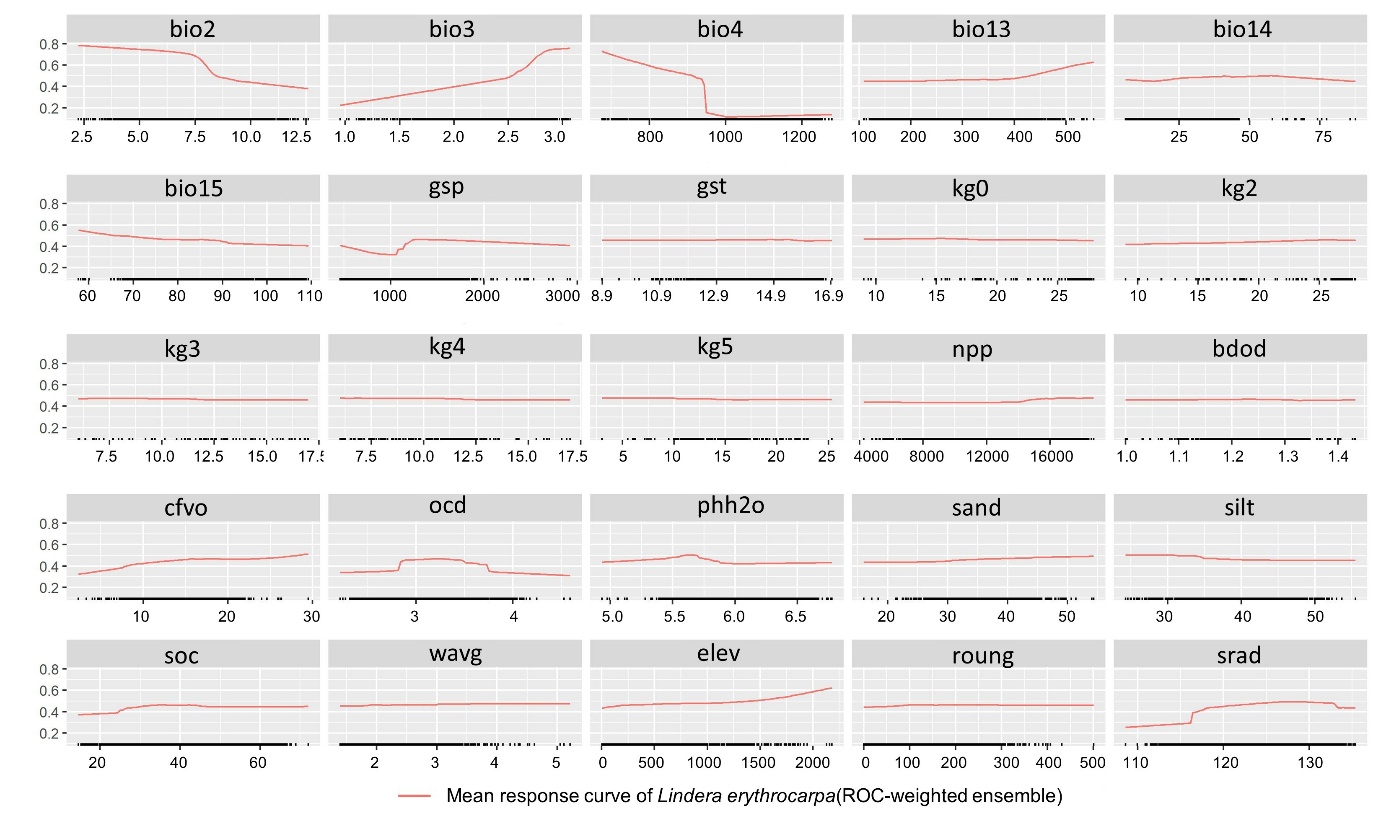
**

S1 Fig. Response curves for environmental variables in the *L. erythrocarpa* ensemble model (six algorithms combined).

**
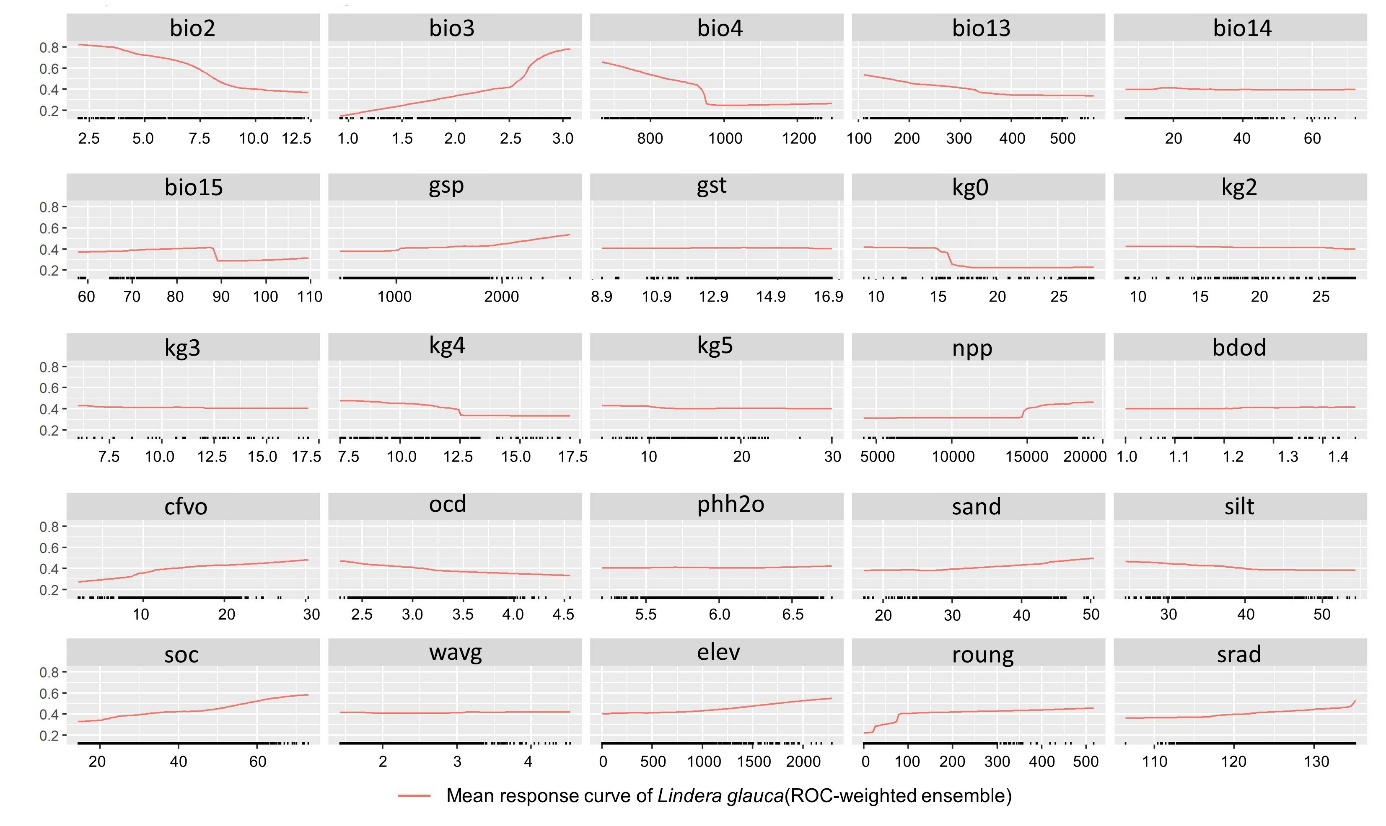
**

S2 Fig. Response curves for environmental variables in the *L. glauca* ensemble model (six algorithms combined).

**
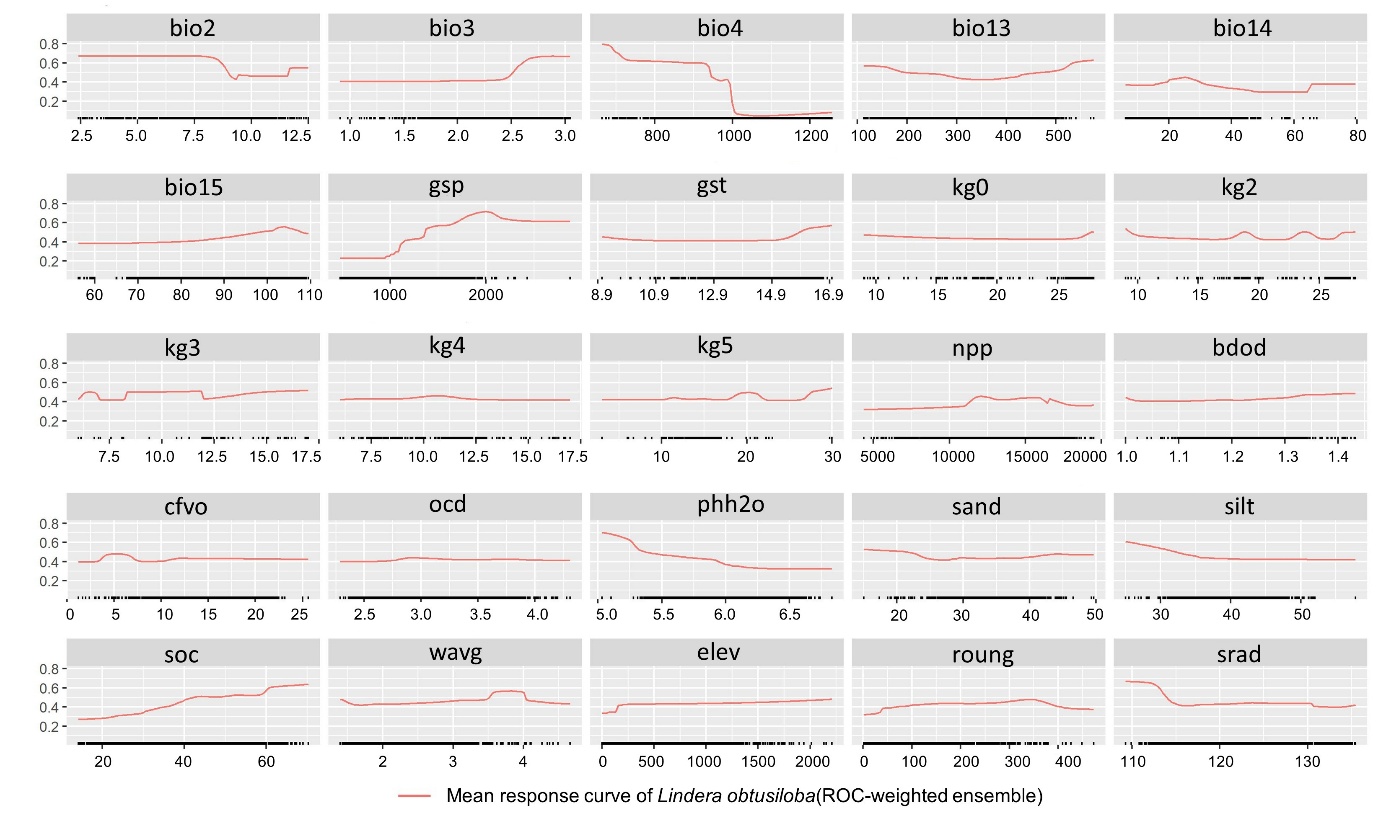
**

S3 Fig. Response curves for environmental variables in the *L. obtusiloba* ensemble model (six algorithms combined).

**
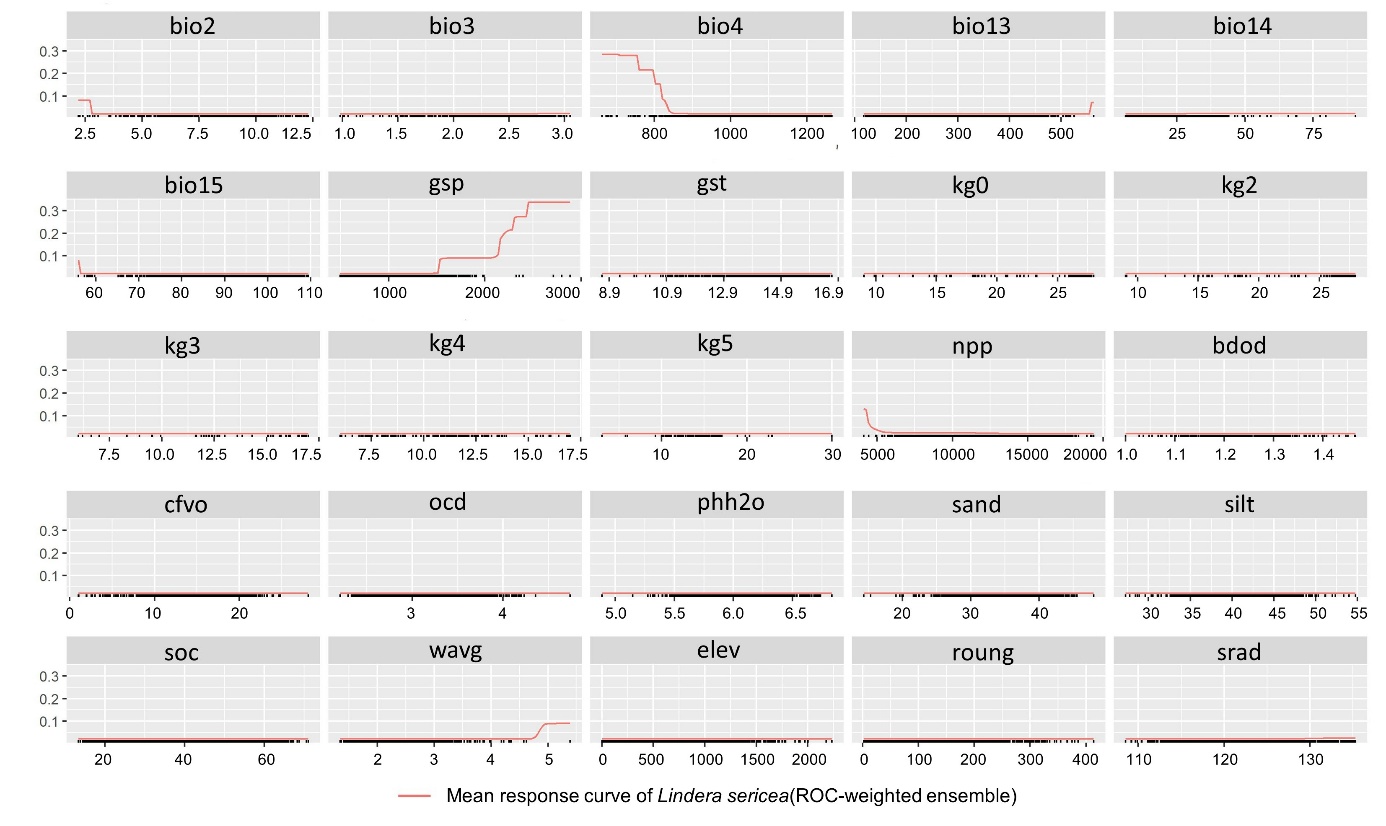
**

S4 Fig. Response curves for environmental variables in the *L. sericea* ensemble model (six algorithms combined).

**
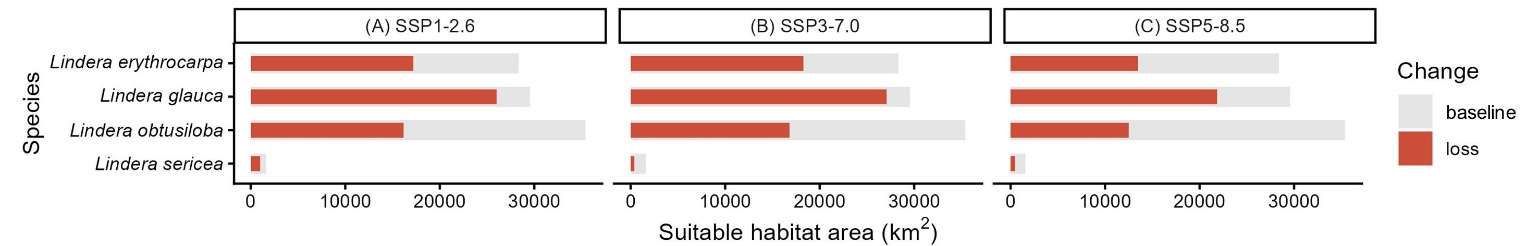
**

S5 Fig. Projected suitable habitat area (km²) of four *Lindera* species under future climate scenarios. Grey bars represent the baseline suitable habitat area, while colored bars indicate projected future areas under each scenario. Colors denote the direction of change relative to the baseline (increase: aquamarine; decrease: red).


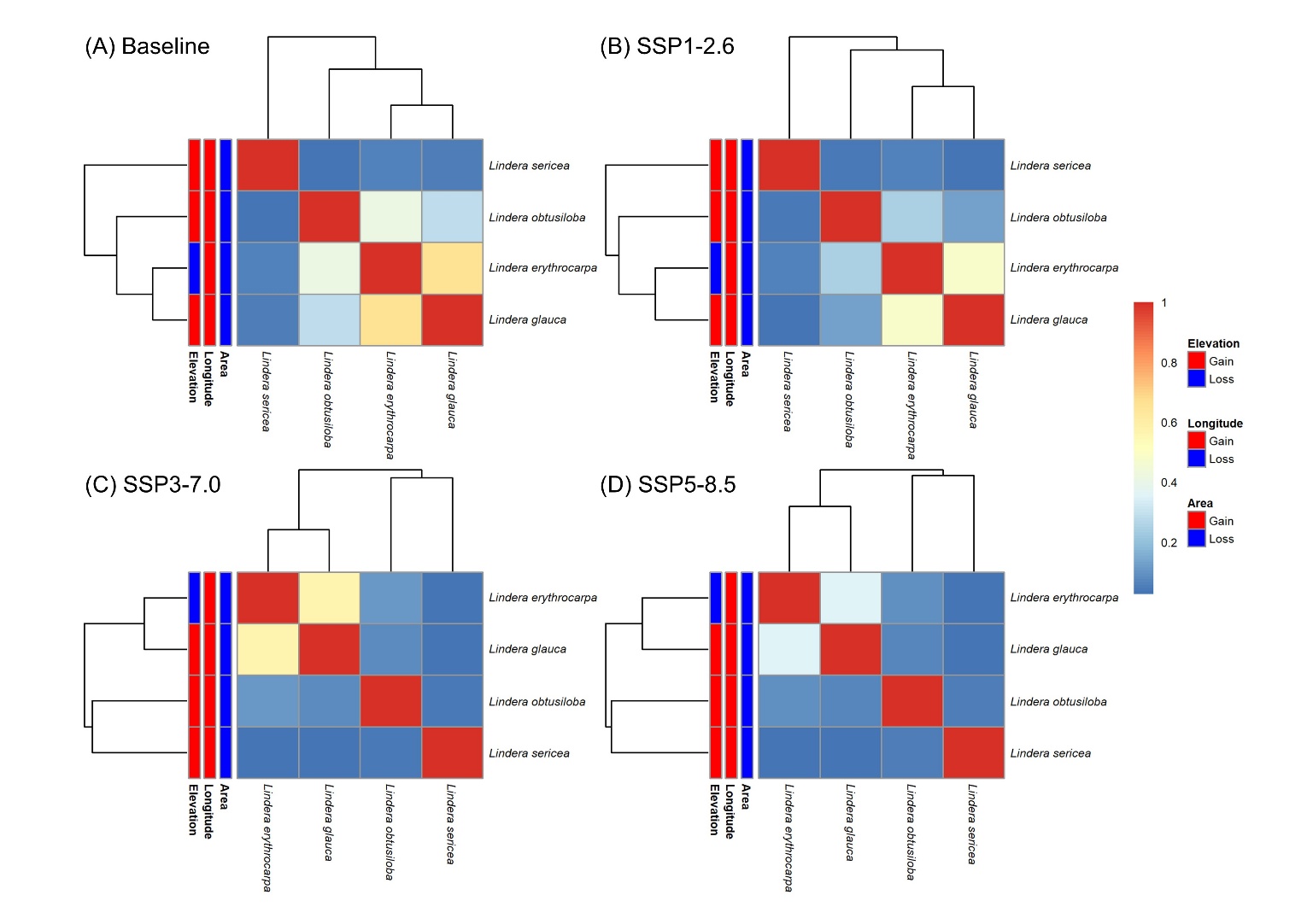


S6 Fig. Niche overlap heatmap of *Lindera* genus under SSP scenarios.

**S1 Table. Environmental variables used in the SDMs, including original spatial resolution and data sources.**

| **Order** | **Class** | **Variable** | **Data source** | **Original spatial resolution** |
| --- | --- | --- | --- | --- |
| 1 | Climate | Annual mean temperature (bio1) | 30 arc-sec  (~1 km) | CHELSA  database (www.chelsa-climate.org) |
| 2 |  | Mean diurnal air temperature range (bio2) |  |  |
| 3 |  | Isothermality (bio2/bio7)(×100) (bio3) |  |  |
| 4 |  | Temperature Seasonality (standard deviation ×100) (bio4) |  |  |
| 5 |  | Max temperature of warmest month (bio5) |  |  |
| 6 |  | Min temperature of coldest month (bio6) |  |  |
| 7 |  | Temperature annual range (bio7) |  |  |
| 8 |  | Mean temperature of wettest quarter (bio8) |  |  |
| 9 |  | Mean temperature of coldest quarter (bio9) |  |  |
| 10 |  | Mean temperature of warmest quarter (bio10) |  |  |
| 11 |  | Mean temperature of coldest quarter (bio11) |  |  |
| 12 |  | Annual precipitation (bio12) |  |  |
| 13 |  | Precipitation amount of the wettest month (bio13) |  |  |
| 14 |  | Precipitation amount of the driest month (bio14) |  |  |
| 15 |  | Precipitation seasonality (bio15) |  |  |
| 16 |  | Precipitation of wettest quarter (bio16) |  |  |
| 17 |  | Precipitation of driest quarter (bio17) |  |  |
| 18 |  | Precipitation of warmest quarter (bio18) |  |  |
| 19 |  | Precipitation of coldest quarter (bio19) |  |  |
| 20 |  | Köppen-Geiger climate classification |  |  |
| 21 |  | Modified Köppen-Geiger climate classification 1 |  |  |
| 22 |  | Modified Köppen-Geiger climate classification 2 |  |  |
| 23 |  | Modified Köppen-Geiger climate classification 3 |  |  |
| 24 |  | Modified Köppen-Geiger climate classification 4 |  |  |
| 25 |  | Precipitation sum accumulated on all days during the growing season based on TREELIM (Paulsen and Körner, 2014) |  |  |
| 26 |  | Mean temperature of all growing season days based on TREELIM |  |  |
| 27 |  | Net primary productivity |  |  |
| 28 | Soil | Bulk density of the fine earth fraction | 250m | SoilGrids  database (www.soilgrids.org) |
| 29 |  | Volumetric fraction of coarse fragments (>2 mm) |  |  |
| 30 |  | Organic carbon density |  |  |
| 31 |  | Soil pH |  |  |
| 32 |  | Proportion of sand particles (> 0.05/0.063 mm) in the fine earth fraction |  |  |
| 33 |  | Proportion of silt particles (≥0.002 mm and ≤ 0.05/0.063 mm) in the fine earth fraction |  |  |
| 34 |  | Soil organic carbon content in the fine earth fraction |  |  |
| 35 | Topographic | Topographic wetness index (TWI) | 90m | NASA SRTM  90 data (www.cmr.earthdata.nasa.gov) |
| 36 |  | Elevation |  |  |
| 37 |  | Surface roughness |  |  |
| 38 |  | Surface solar radiation |  |  |

**S2 Table. Predictive performance of individual algorithms used in the SDMs, evaluated using Cohen’s Kappa, the area under the receiver operating characteristic curve (ROC), and the true skill statistic (TSS).**

| **Algorithm** | **Kappa (mean ± SD)** | **ROC (mean ± SD)** | **TSS (mean ± SD** |
| --- | --- | --- | --- |
| ANN | 0.512 ± 0.109 | 0.952 ± 0.034 | 0.729 ± 0.122 |
| CTA | 0.609 ± 0.126 | 0.878 ± 0.043 | 0.703 ± 0.109 |
| GAM | 0.644 ± 0.096 | 0.904 ± 0.035 | 0.681 ± 0.105 |
| GBM | 0.718 ± 0.112 | 0.941 ± 0.038 | 0.743 ± 0.094 |
| MARS | 0.652 ± 0.097 | 0.908 ± 0.038 | 0.689 ± 0.093 |
| RF | 0.719 ± 0.109 | 0.951 ± 0.033 | 0.712 ± 0.076 |

**S3 Table. Optimal threshold statistics of the ensemble model (EMwmean), including sensitivity, specificity, and cutoff values calculated for Kappa, ROC, and TSS metrics.**

| **Algorithm** | **Metric** | **Average sensitivity (%)** | **Standard deviation of sensitivity** | **Average specificity (%)** | **Standard deviation of specificity** | **Average cutoff** | **Standard deviation of cutoff** |
| --- | --- | --- | --- | --- | --- | --- | --- |
| EMwmean | KAPPA | 90.7 | 5.6 | 94.1 | 3.4 | 619.8 | 72.0 |
| EMwmean | ROC | 94.2 | 3.7 | 91.6 | 4.7 | 567.5 | 41.7 |
| EMwmean | TSS | 94.0 | 3.7 | 91.9 | 4.5 | 572.4 | 45.3 |
